# Local Disease-Free Survival and Disease-Free Survival in Locally Advanced Cervical Cancer Diagnosed and Treated in Bihor County, Romania

**DOI:** 10.7759/cureus.65629

**Published:** 2024-07-29

**Authors:** Ottó Molnar, Simona Mihuțiu, Codrin D Ilea, Alexandra Vesa, Oreste M Straciuc, Noémi Németh, Liviu Lazăr

**Affiliations:** 1 Doctoral Studies Department, Biomedical Science, University of Oradea, Oradea, ROU; 2 Department of Medicine-Psycho-Neuroscience and Recovery, University of Oradea, Oradea, ROU; 3 Statistics, Bihor County Emergency Clinical Hospital, Oradea, ROU; 4 Faculty of Medicine and Pharmacy, Morphological Sciences, University of Oradea, Oradea, ROU

**Keywords:** neoadjuvant chemotherapy, locally advanced cervical cancer (lacc), disease-free survival, local disease-free survival, tumor grading, surgery, cervical cancer

## Abstract

Introduction: Cervical cancer is the fourth most dangerous malignancy worldwide in women and is diagnosed at the advanced stages in most cases. Oncological and surgical modalities when precisely employed together can prove to be helpful for determining the proper diagnosis and treatment strategies. Neoadjuvant chemotherapy (NACT) has been found to have a role in reducing tumor size and has evolved as a treatment regimen for locally advanced cervical cancer (LACC). The present study aimed to analyze the treatment strategies either with neoadjuvant platinum-based chemotherapy (NACT) administration or not and pathological responses in patients with LACC.

Methods: We reviewed 100 patients of LACC from October 2018 to December 2022 at Bihor County Emergency Clinical Hospital. About 43 patients underwent radiation therapy in addition to NACT administration (NACT+/other) and 57 underwent other treatment regimens without neoadjuvant treatment (NACT-/ other). Various demographic parameters, FIGO staging, histological status, surgical interventions, and survival rate (local disease-free survival (LDFS) and disease-free survival (DFS)) were accessed in both groups. Statistical analysis was performed to analyze the significance of various parameters studied.

Results: The mean age range of the studied sample was 57.05 ± 12.51 in NACT+/other and 60.4±12.32 in NACT-/other. Among 100 patients, 90 cases of squamous carcinoma, eight of adenocarcinoma, and two cases of adenosquamous carcinoma were analyzed and treated. At stage IIIC1, 11 patients were accessed while 15 patients were at clinical stage IIIC2, and among these, 25.58% received neoadjuvant oncological treatment and very limited mediastinal disease. DFS rates are greater in the patients who have undergone surgery in the NACT+/other group, while in the LDFS, there is better survival in the case of surgery without any NACT treatment (NACT-/other).

Conclusions: The effect of NACT can be suggested as another important treatment strategy and result in a good response in terms of DFS and LDFS in patients with LACC. This approach aims to reduce tumor size preoperatively, facilitating surgical removal and potentially improving patient outcomes compared to other treatment modalities. Thus, it can be concluded that NACT may be considered an important strategy to be opted for the treatment of LACC.

## Introduction

Cervical cancer is predominantly caused by the high-risk strains of human papillomavirus (HPV), which account for around 70% of cervical cancers worldwide. Mortality rates vary dramatically, with disparities in different regions depending on various factors. These significant differences in cervical cancer outcomes are likely influenced by factors such as access to healthcare, the presence and effectiveness of screening programs, and the distribution and utilization of HPV vaccines, which are considered to be the key factors in preventing cervical cancer [1]. Despite these advancements, cervical cancer remains a significant challenge, with treatment paradigms still primarily focused on the complete eradication of cancer cells.

Despite cervical cancer being a largely preventable disease, the situation in Romania is particularly concerning [2]. Following colorectal cancer and breast cancer, cervical cancer ranks as the third most common cancer among women in Romania. According to the World Health Organization (WHO), Romania is found to have the highest rate of mortality from cervical cancer among European Union countries [3]. Out of all cervical cancer cases diagnosed in the EU, about 7.5 % occur in Romania, which is almost three times higher than in the EU [4]. The impact of cervical cancer screening has been profound, with a dramatic reduction in incidence and mortality observed between the 1960s and 1990s, credited to the widespread adoption of the Pap test [5,6]. Accurate staging is crucial to determine the precise extent of cancer, as failing to identify disease outside the radiation field can lead to under-treatment, whereas incorrect staging could result in unnecessary exposure to radiation or other therapies.

Noninvasive imaging techniques, such as computed tomography (CT) and magnetic resonance imaging (MRI), are routinely used to evaluate the extent of local advancement and identify any metastasis. These imaging modalities are fundamental in guiding treatment decisions to ensure the optimal balance between effectiveness and safety. Stage IIIA disease, which extends to the lower one-third of the vagina, is best evaluated using a sagittal sequence. Involvement of lymph nodes in the staging classification results in upstaging for a significant number of patients, thereby more accurately reflecting their prognosis. Notably, there is an observed increase in the survival rates of five years in patients with stage IIIC1 disease compared to those with stage II disease. Stage IV disease encompasses rectal and bladder mucosal involvement, as well as metastases to the inguinal and supraclavicular lymph nodes, lungs, bones, and other visceral organs. Patients at the metastatic stage are then referred for definitive oncological treatment, which utilizes various strategies such as chemotherapy and radiotherapy, as well as immunological therapies [7].

Neoadjuvant chemotherapy (NACT) has an effective role in decreasing the risk of different pathological issues linked to cervical cancer prognosis. Cervical carcinoma, when confined to the lower pelvis, can be treated successfully through surgery or chemoradiotherapy. For women with tumors smaller than 2 cm that do not involve the uterus, transvaginal radical trachelectomy offers a fertility-preserving option. However, surgery alone is inadequate for women with lymphatic metastases, and pelvic irradiation is ineffective if metastases extend beyond the irradiated area. Treatment decisions thus rely on secondary indicators such as tumor size and its spread to the cervical stroma, uterine corpus, or parametrium. Enhanced imaging for better tumor delineation aids in these decisions and improves the accuracy of targeted radiation therapy. Recent increases in cancer survival rates can be attributed to improvements in screening and advancements in treatment methods. Efforts to enhance cervical cancer survival have concentrated on early detection through screening and the prevention of the disease via HPV vaccination. Studies indicate that combining radiation therapy with chemotherapy is more effective than radiation therapy alone. Specifically, the five-year survival rate for patients with stage IB, IIA, or IIB cervical cancer is 77% with concurrent chemoradiotherapy, compared to 50% with radiation therapy alone. This combination therapy is generally well tolerated, with only minor and reversible gastrointestinal and hematologic side effects [8-16]. In the present retrospective study, various demographic parameters were taken into account, and various surgical procedures after NACT or not and their effects on survival rates were analyzed.

## Materials and methods

Study design and patient data

A retrospective study was conducted on 100 patients diagnosed with locally advanced cervical cancer (LACC). One group received NACT in addition to standard radiotherapeutic procedures (NACT + radiotherapy/radiochemotherapy/brachytherapy). The other group was not administered NACT and just employed other treatment strategies (NACT- radiotherapy/radiochemotherapy/brachytherapy). Diagnostics and treatment (radiotherapy, chemotherapy, and surgery) were performed at Bihor County Emergency Clinical Hospital.

Inclusion and exclusion criteria

The subjects who were selected and included in the study were residents of Bihor County (≤18 years) based on the histopathological diagnosis of cervical cancer. From October 1, 2018, to December 31, 2022, the patients were at a locally advanced stage of the disease according to the FIGO 2018 staging (stages IIB, III, and IVA). The patients with hysterectomy with bilateral salpingo-oophorectomy (surgery to remove both of your ovaries and fallopian tubes) with lymphadenectomy or hysterectomy (lymph node dissection) performed as the primary treatment. Receipts of other treatments, whether systemic or local and outside of Bihor County, were excluded from the study. Cervical biopsies were performed by gynecologists in the healthcare institutions of Bihor County. Only patients who gave their consent for participation were included in the study. The patients who received hysterectomy with bilateral salpingo-oophorectomy and lymphadenectomy as primary treatment were also excluded from the study.

Clinical diagnosis

The signs and symptoms reported by patients were documented in the observation sheets/discharge summaries of the gynecology departments. Cervical examination with speculum was documented in the observation sheets. Definitive diagnosis and histopathological examination were conducted by an expert pathologist. In both groups under study, with diverse symptoms of the disease, a gynecological examination was performed, followed by biopsy sampling from the cervix under anesthesia. CT scans of the chest, abdomen, and pelvis were performed in all cases, and in cases without contraindications for contrast material, this method was preferred. In situations where patients had multiple chronic comorbidities, advanced age, or vaginal bleeding not requiring immediate intervention, pelvic MRI was not performed. Those patients with suspected distant metastases, suspicious lymphadenopathy, or advanced local invasion beyond what was described by CT and MRI were further evaluated with PET-CT using fluorodeoxyglucose (FDG). Disease staging was performed using either CT, MRI, or PET-CT, and the FIGO 2018 classification was utilized.

Treatment regimens

Patients underwent systemic treatment in the Oncology Department or the Day Oncology Hospitalization Unit of the Bihor County Emergency Clinical Hospital. Each patient included in the study received 3D conformal external beam radiation therapy. In terms of surgical approach, 31 patients received surgical treatment. At the radiotherapy phase at 46-50 GY, a gynecological examination was done, and if a good clinical response was found with the reduction of the tumor, patients were treated surgically. Definite chemoradiation was administered at 60 GY in the case of no treatment response. Two types of surgical procedures were performed: Wertheim operation and hysterectomy without lymphadenectomy. At an irradiation of 46-48 GY, Wertheim operation was preferred, and at 50 GY or above, hysterectomy without lymphadenectomy was opted for.

Statistical analysis

The analysis was performed by formulating the null statistical hypothesis of whether NACT administration has any effect on therapeutic decisions and survival patterns. For testing the hypotheses, a series of tests were applied, depending on the type of variable analyzed. In the case of continuous variables, the t-test (Student's t-test) chi-square test (χ²) and the ANOVA test were applied. Survival analysis was performed using the Kaplan-Meier survival curve method.

## Results

**Figure 1:**
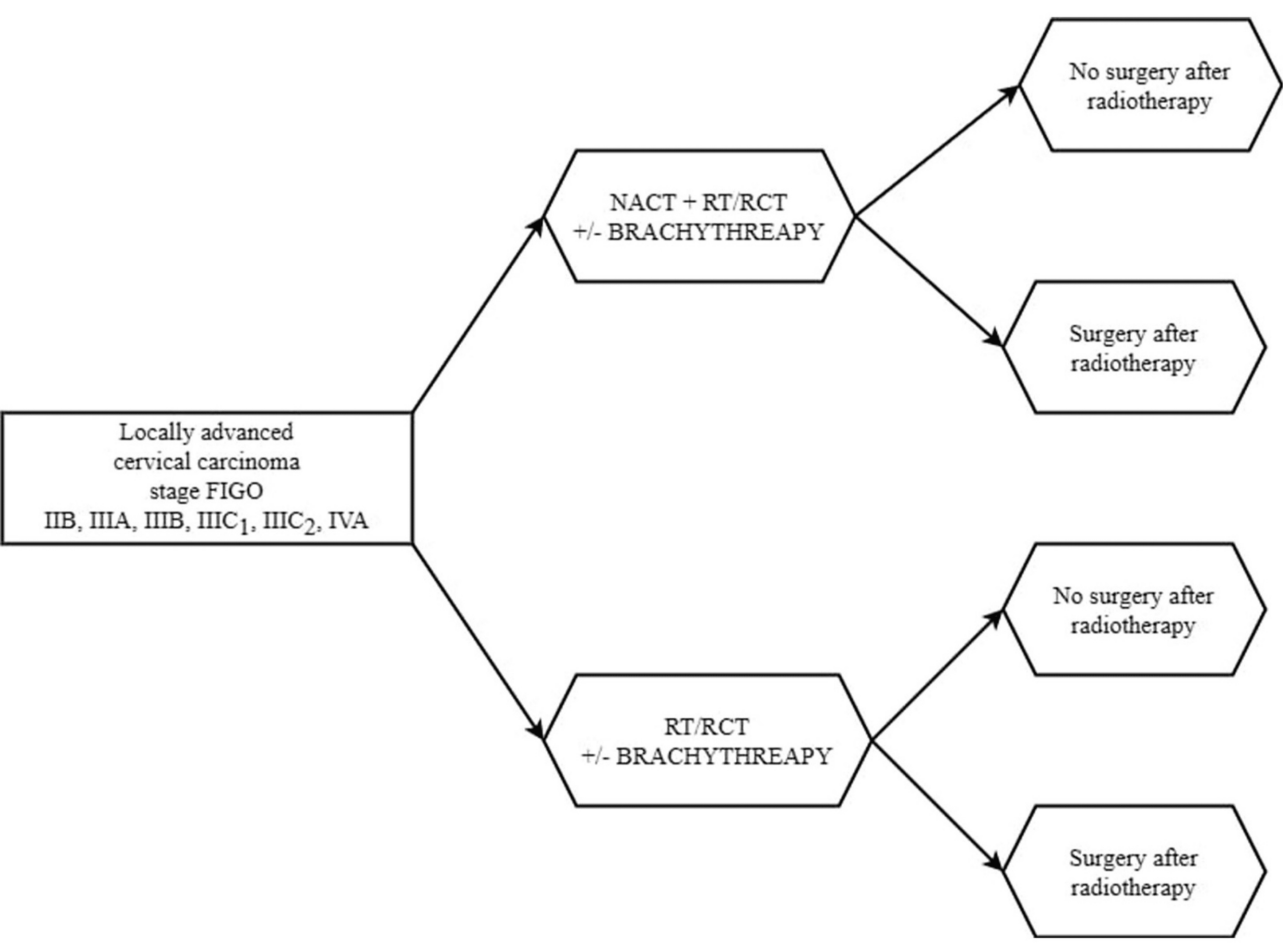
Various treatment strategies employed at various stages according to the FIGO system FIGO: International Federation of Gynecology and Obstetrics

A hundred patients diagnosed with LACC from October 1, 2018, to December 31, 2022, were included in the study. The study group (NACT+/other) comprised 43 cases who received neoadjuvant chemotherapy plus other treatment means, i.e., radiotherapy, radiochemotherapy, or brachytherapy, and the control group (NACT-/other) received only standard treatment without neoadjuvant chemotherapy (Figure 1).

The mean age range was 57.05 ± 12.51 in the NACT+/other group and 60.4 ± 12.32 in the NACT-/other group. Cases who received neoadjuvant therapies prevalently belong to the rural settlements. Diseases such as hypertension were found to be significantly associated with patients with cancer, whereas chronic ischemic heart disease, atrial fibrillation, and venous thrombosis were found to have a non-significant association (Table 1). The comparison of age between the two groups was tested by a T-test, whereas residential status was analyzed by the chi-square test. The statistical data analysis was performed on 100 patients.

**Table 1:** Demographic parameters and prevalence of associated cardiovascular diseases in the studied patients

Characteristics	Total cases	NACT + other	NACT- other	p value
No. of patients n (%)	100	43	57	---
Age				
Medium age	58.96	57.05	60.4	0.2
Standard deviation (SD)	12.45	12.51	12.32	
Residence				
Rural	50	23 (53.49%)	27 (47.37%)	0.7
Urban	50	20 (46.51%)	30(52.63%)	
Associated symptoms				
Hypertension				
No	58	30 (69.77%)	28 (49.12%)	0.06
Yes	42	13 (30.23%)	29 (50.88%)	
Chronic ischemic heart disease				
No	79	34 (79.07%)	45 (77.59%)	1
Yes	21	9 (20.93%)	13 (22.41%)	
Atrial fibrillation				
No	96	41 (95.35%)	55 (96.49%)	1
Yes	4	2 (4.65%)	2 (3.51%)	
Venous thrombosis				
No	98	42 (97.67%)	56 (98.25%)	1
Yes	2	1 (2.33%)	1 (1.75%)	

The maximum number of patients were assessed as clinical stage IIIB. Among the two groups (NACT+/other and NACT-/other), the clinical stage was significantly different. Among 36 patients with stage III B cervical cancer, 15 went through NACT and 21 underwent other treatment methods without neoadjuvant therapy. At stage IIIC1, 11 patients were accessed while 15 patients were at clinical stage IIIC2, and among these, 25.58% received neoadjuvant oncological treatment and very limited mediastinal disease (Table 2).

**Table 2:** Classification of patients under study based on the standard FIGO staging FIGO: International Federation of Gynecology and Obstetrics

FIGO Stage	Total	NACT +/ other	NACT-/ other
II B	20	3 (6.98%)	17 (29.82%)
III A	7	1 (2.33%)	6 (10.53%)
III B	36	15(34.88%)	21 (36.84%)
III C1	11	7 (16.28%)	4 (7.02%)
III C2	15	11 (25.58%)	4 (7.02%)
IV A	11	6 (13.95%)	5 (8.77%)
Total	100	43	57
			p < 0.01

Of 100 cases, 74 had squamous cell carcinoma, and 14 had papillary squamous carcinoma. Five patients had adenosquamous cell carcinoma, two had mucinous adenocarcinoma, two had adenosquamous carcinoma, one had papillary serous adenocarcinoma, one had squamous cell carcinoma, and one had squamous spinocellular carcinoma (Table 3). Sixty-six patients were found at tumor grade II, and among these, 71.43% received NACT in addition to other treatment regimens, while 65.45% were treated without NACT. Similarly, 29.09% of the patients did not receive NACT among patients suffering from stage III cervical cancer. It can be concluded that the patients at all tumor grades were in the NACT- group. The radiation equivalent doses of >85 Gy in were 86% and 13.95% in the NACT+/other and NACT-/other groups, respectively. In the NACT- group, 17.54% of the patients had radiation doses greater than 85 GY as compared to the NACT+ group (13.95%; <85 GY dose).

**Table 3:** Various cervical cancer types in patients and their grading

Cancer types	Total cases	NACT +/other	NACT-/ other
No of Patients n (%)	100	43	57
Squamous			
Squamous cell carcinoma	74	32(74.418%)	42(73.6842%)
Basaloid squamous cell carcinoma	1	1(2.3255%)	0
Papillary Squamous Carcinoma	14	5(11.6279%)	9(157894%)
Squamous Spinocellular Carcinoma	1	1(2.3255%)	0
Non-squamous / Adenocarcinoma			
Adenocarcinoma	5	2(4.6511%)	3(5.263%)
Mucinous adenocarcinoma	2	1(2.3255%)	1(1.7543%)
Papillary Serous Adenocarcinoma	1	1(2.3255%)	0
Adeno squamous carcinoma	2	0	2(3.5087%)
GRADING*			
I	4	1 (2.38%)	3 (5.45%)
II	66	30 (71.43%)	36 (65.45%)
III	27	11 (26.19%)	16 (29.09%)
Equivalent doses			
>85GY	84	37 (86.04%)	47 (13.95%)
<85GY	16	6 (13.95%)	10 (17.54%)
			p= 0.8342

For the surgical treatment in terms of surgical approach, a total of 31 patients received surgical treatment. Each patient was given radiotherapeutic treatment, and among these, eight patients received surgery after radiation therapy and NACT administration (NACT+/other), and 23 were surgically intervened and received radiation therapy without any neoadjuvant administration (NACT-/other). Six patients recevied surgery by hysterectomy with bilateral anexectomy, and in 22 patients, lymphadenectomy in addition to hysterectomy with bilateral anexectomy was done. The surgical procedure was started in three cases that had a partial response at clinical examination but the disease seemed bulky and so messed up that surgery was not done finally. One case had a colostomy and hysterectomy, and a recto-sigmalfistulation was done prior to treatment. The number of patients who underwent surgical treatment without NACT was greater than the group who received NACT (Table 4).

**Table 4:** Various surgical procedures adopted in the patients after NACT or without NACT NACT: neoadjuvant chemotherapy

Surgical procedures	Total cases	Surgery (NACT+ and radiotherapy)	Surgery (NACT- and radiotherapy)
Number	100	43	57
No surgery	69	35 (81.40%)	34 (59.65%)
Surgery with intervention	31	8 (18.60%)	23 (40.35%)
			p = 0.03491
Surgery type			
Total hyesterectomy with bilateral anexectomy	6	1 (2.33%)	5 (8.77%)
Total hyesterectomy with bilateral anexectomy + lymphadenectomy	22	5 (11.63%)	17 (29.82%)
Other situations*	3	2 (4.65%)	1 (1.75%)
			p = 1

In patients with early-stage cervical cancers, it was found that patients who underwent NACT had better local disease-free survival (LDFS) (IIB, 95%CI = 27.4-97.4, p = 0.58) up to 35 months compared to those without NACT administration (IIB, 95%CI = 24.14-39.51, p = 0.58). LDFS up to 32 months was observed in patients suffering from stage IIIB (95%CI = 22.38-42.02, p = 0.58) in patients administered with NACT. The same group without NACT showed survival rates up to around 23 months. The survival rates decreased in each group at advanced diseased stages. Thus, it can be concluded that in terms of LDFS, administering NACT before standard treatment regimens is found more effective in locally advanced cervical cancer as compared to the group without NACT (Table 5). In terms of disease-free survivals (DFS), patients with early or locally advanced cervical cancer found that patients who underwent NACT in addition to radiation therapy had better DFS at stage IIB (95%CI = -27.4-97.4, p = 0.37) up to 35 months compared to those who without NACT (95%CI = 21.25-38.87, p = 0.37) (Table 6).

**Table 5:** Average LDFS rates in patients who underwent radiation therapy with NACT or without NACT LDFS: local disease-free survival, NACT: neoadjuvant chemotherapy, FIGO: International Federation of Gynecology and Obstetrics, SD: standard deviation, CI: confidence interval

FIGO stage	Total cases	NACT+/ other (n)	Average value for LDFS (in months)	SD	CI 95%	NACT-/ other (n)	Average value for LDFS (in months)	SD	CI 95%
II B	20	3	35.00	25.12	>> 27.4 <> 97.4	17	31.82	14.95	24.14 <> 39.51
III A	7	1	43.00			6	47.00	11.87	34.55 <> 59.45
III B	36	15	32.20	17.73	22.38 <> 42.02	21	23.29	13.47	17.15 <> 29.42
III C1	11	7	22.00	17.93	5.42 <> 38.58	4	16.75	11.67	>> -1.82 <> 35.32
III C2	15	11	15.45	12.88	6.80 <> 24.11	4	13.50	8.27	0.35 <> 26.65
IV A	11	6	22.33	20.40	0.92 <> 43.74	5	14.40	11.80	>> -0.25 <> 29.05
Total	100	43				57			p= 0.58

**Table 6:** Average DFS rates in patients who underwent radiation therapy with NACT or without NACT LDFS: local disease-free survival, NACT: neoadjuvant chemotherapy, FIGO: International Federation of Gynecology and Obstetrics, SD: standard deviation, CI: confidence interval

FIGO stage	Total cases	NACT+/ other (n)	Average value for DFS (in months)	SD	CI 95%	NACT-/ other (n)	Average value for DFS (in months)	SD	CI 95%
II B	20	3	35.00	25.12	>> -27.4 <> 97.4	17	30.06	17.14	21.25 <> 38.87
III A	7	1	43.00			6	47.00	11.87	34.55 <> 59.45
III B	36	15	32.20	17.73	22.38 <> 42.02	21	22.43	14.35	15.90 <> 28.96
III C1	11	7	16.86	17.77	0.42 <> 33.29	4	16.75	11.67	>> -1.82 <> 35.32
III C2	15	11	15.18	13.15	6.35 <> 24.02	4	10.50	7.23	>> -1.01 <> 22.01
IV A	11	6	20.67	21.87	>> -2.28 <> 43.62	5	14.40	11.80	>> -0.25 <> 29.05
Total	100	43				57			p= 0.37

**Figure 2:**
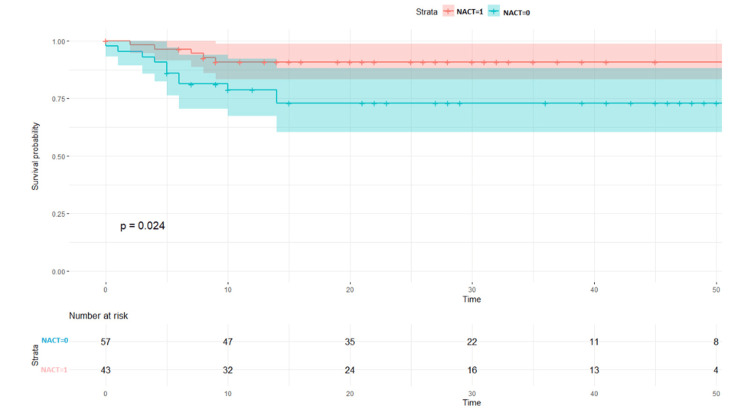
Local disease-free survival rates between the NACT+ and NACT- groups NACT: neoadjuvant chemotherapy

**Figure 3:**
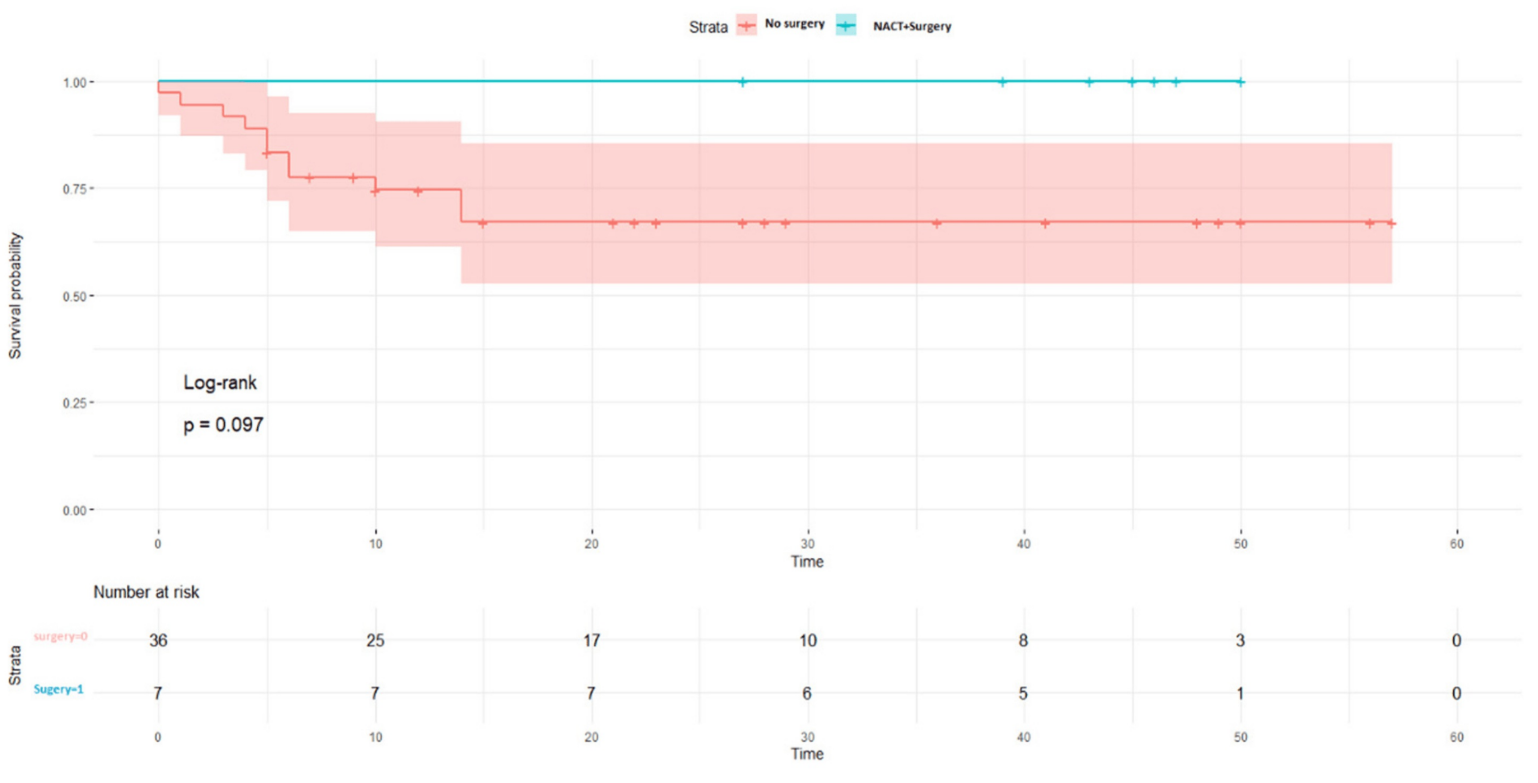
Local disease-free survival rates with surgery in the NACT+ groups NACT: neoadjuvant chemotherapy

**Figure 4:**
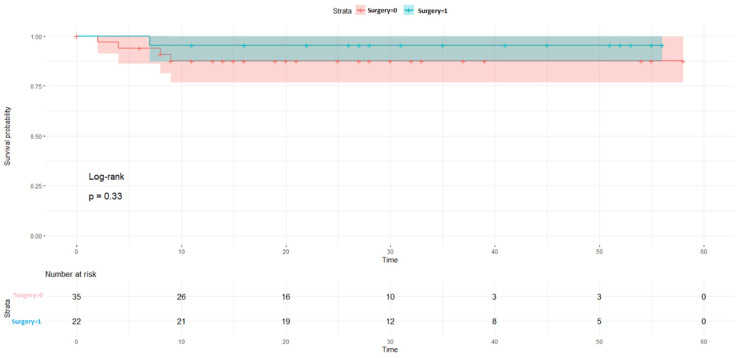
Local disease-free survival rates with surgery in the NACT- groups NACT: neoadjuvant chemotherapy

The LDFS survival rates were 87% in the NACT +/other group and 73% in the NACT-/other group (p = 0.024, Figure 2). When the surgical procedure was performed, it was found that a five-year survival rate of 100% was observed in the patient NACT+/other surgery group (p = 0.097, Figure 3). LDFS rates were reduced in patients in which surgery was done without any neoadjuvant chemotherapy (NACT-) group (Figure 4). NACT+/other surgery was found to be an independent risk prognostic factor for LDFS (95%CI = 27.4-97.4, p = 0.58), as shown in Table 5.

**Figure 5:**
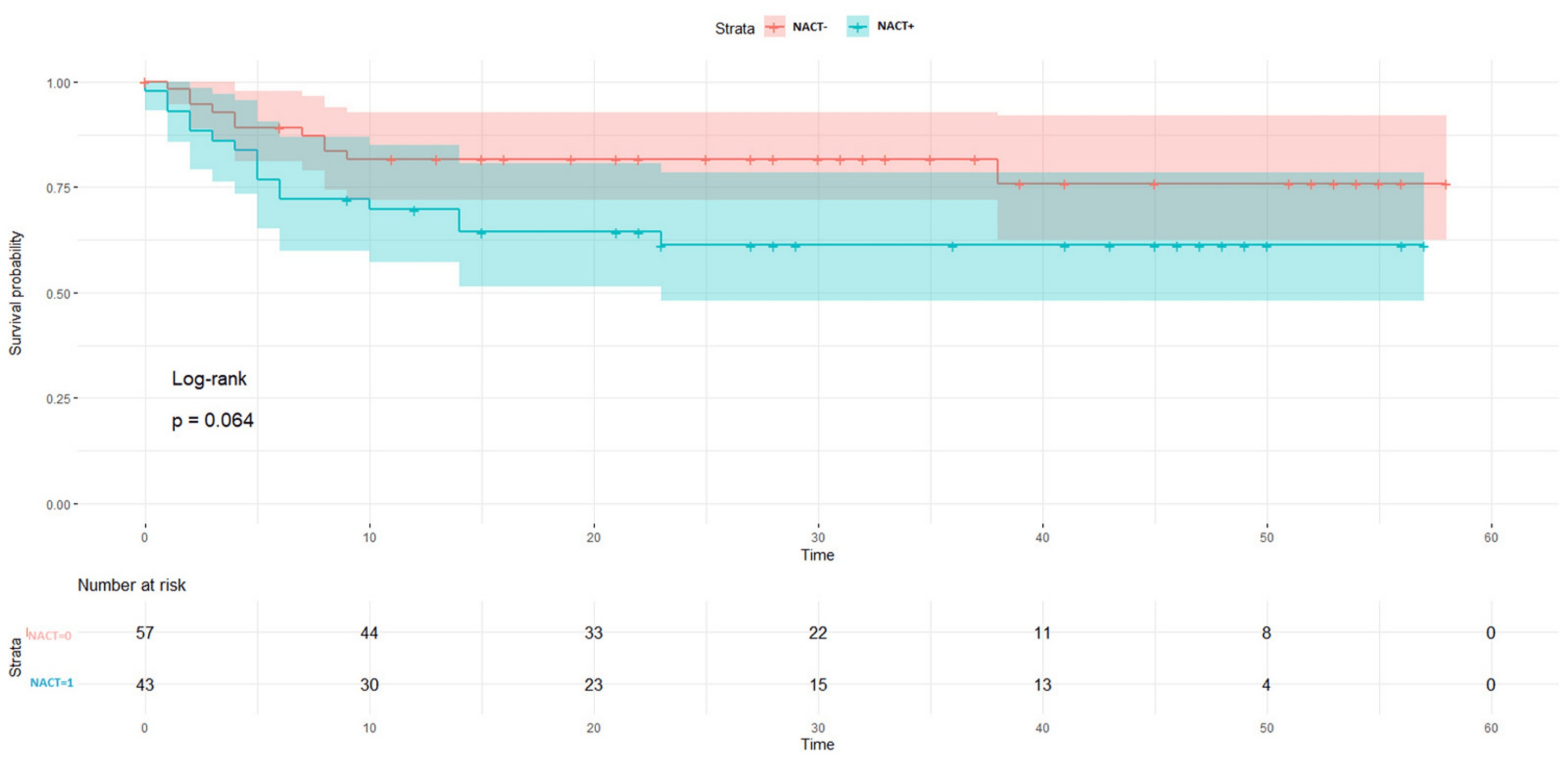
Disease-free survival rates between the NACT+ and NACT- groups NACT: neoadjuvant chemotherapy

**Figure 6:**
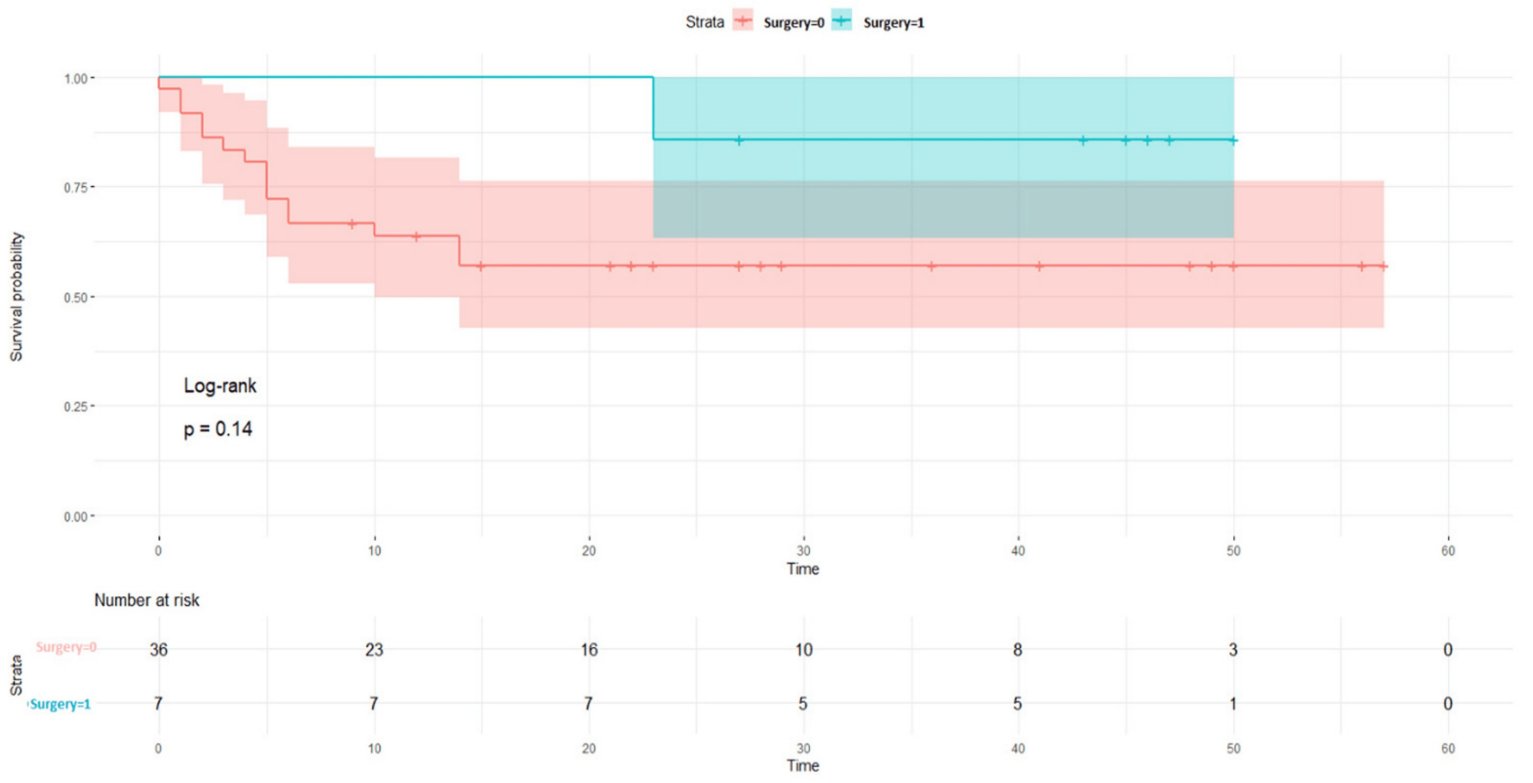
Disease-free survival rates with surgery in the NACT+ groups NACT: neoadjuvant chemotherapy

**Figure 7:**
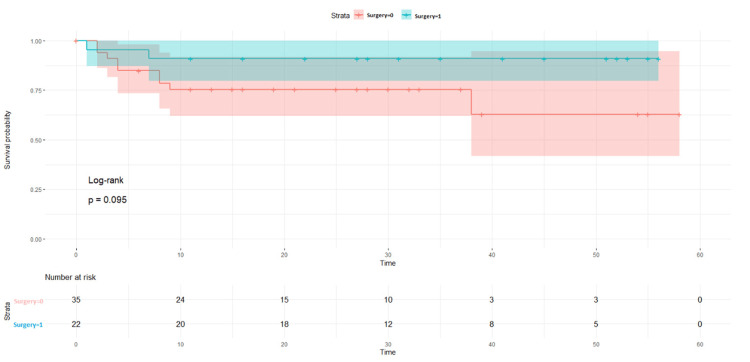
Disease-free survival rates with surgery in the NACT- groups NACT: neoadjuvant chemotherapy

Before the surgery intervention, DFS survival probability was found to be greater in the NACT -/other group as compared to others, i.e., p = 0.002 (Figure 5). When surgery was performed, in the NACT+/other surgery group, the DFS ratio was found 89% (p = 0.14) as compared to the NACT-/other surgery, which is 83% (p = 0.095) (Figures 6, 7).

## Discussion

Among various cell carcinomas, squamous cell carcinoma accounts for approximately 90% of cervical cancer cases and develops from the squamous epithelial cells of the ectocervix. Staging information is crucial for clinicians to create individualized treatment plans that address the specific characteristics of the disease while minimizing the risks of overtreatment or undertreatment. The FIGO classification system is universally adopted for defining the stages of cervical cancer [7]. Several variables were analyzed in our study to identify risk factors affecting survival time, age, equivalent doses administered, FIGO stage, tumor grading, and treatment type. Data on other potential risk factors, such as the age at the first pregnancy, sexual partner’s number, smoking status, Pap smear test history, and tumor histopathology, were not available. Consequently, the accessible variables were used to assess their impact on survival time. The sensitivity of cervical cancer to chemotherapy was demonstrated in 1983 concluding that combining radiotherapy with chemotherapy significantly improves the cervical cancer prognosis, reducing the risk of local or distant relapse as compared to radiotherapy alone.

For early-stage cervical cancer, where the malignancy is confined to the cervix, treatment options typically include radical surgery or radical radiotherapy. Radical surgery involves the removal of the cervix, uterus, fallopian tubes, and potentially other surrounding tissues. Alternatively, radical radiotherapy uses high-energy X-rays to target and destroy cancer cells. Clinical studies have shown that both radical surgery and radiotherapy are equally effective for early-stage cervical cancer. In cases where the tumor is larger or has spread to adjacent tissues (locally advanced disease), the standard treatment approach often includes chemoradiation, which combines radiotherapy with chemotherapy to enhance the effectiveness of the treatment. Since 1999, the use of combined radiotherapy and chemotherapy has been recommended for patients with LACC. Currently, chemotherapy, particularly NACT, is widely utilized in clinical practices for the treatment of cervical cancer [17-19]. The precision of radiation delivery and minimum exposure to surrounding tissues at risk determine the effectiveness of treatment response and further strategies to be employed. In patients suffering from advanced-stage cervical cancer, radiation therapy has been administered as a standard treatment compared to any surgical involvement aiming to cure. Thus, two types of equivalent doses were administered >85 Gy and <85 Gy. Patients who receive greater than 85 Gy as compared to lower doses are found a longer duration of treatment response. The radiation doses were calibrated to minimize the impact on adjacent structures such as the rectum and bladder. To standardize bladder filling and reduce inter-fraction motion, a systematic drinking protocol is mostly implemented, ensuring reproducible bladder volumes, which is critical for maintaining [20].

3D-conformal radiotherapy (3DCRT) provides the improvement of recording volumetric dosimetry and related toxicities and treatment outcomes. In addition, NACT, which is given before radical surgery, may be employed to shrink the tumor, making it easier to remove surgically. This approach can also help eliminate microscopic tumors that are not easily detectable. By reducing the tumor size prior to surgery, NACT may increase the likelihood of complete tumor removal and improve overall prognosis. Overall, the management of cervical cancer varies based on the stage of the disease, with tailored treatment plans aimed at maximizing efficacy and improving survival rates. The prevailing therapeutic regimen for LACC has entailed the administration of radiation therapy concomitantly with cisplatin, yielding a noteworthy 12% enhancement in overall survival rates relative to radiotherapy as a standalone intervention. The imperative to explore more efficacious therapeutic modalities is underscored by the sobering statistic that approximately 40% of afflicted individuals endure disease recurrence within a five-year timeframe [21,22]. Preoperative NACT for cervical cancer was reported by Benedetti et al., who utilized a combination chemotherapy regimen consisting of cisplatin, methotrexate, and bleomycin to treat LACC. The patients showed a 75.7% response rate and all patients who received chemotherapy then went through radical hysterectomy [23]. Finally, NACT before surgery has progressively become a common treatment strategy for cervical cancer. In stage III cervical cancer patients, NACT has found profound potential for surgical procedures and demonstrated certain levels of success. These results are consistent with findings from other studies, supporting the efficacy of NACT in improving surgical outcomes and local DFS and DFS for advanced cervical cancer [24].

However, within the cohort of patients diagnosed with stages IB2 and IIA, a proportion may attain comparable outcomes through the application of NACT followed by surgical intervention in contrast to chemoradiation, as suggested by subgroup analyses derived from the Tata Memorial Hospital study. The surgical method comprised a radical hysterectomy combined with pelvic lymph node dissection [25-27]. The results are in justification that at early stages even without administration of neoadjuvant drugs, surgery can be proven to be beneficial in terms of survival rates. In a more contemporary context, the utilization of NACT preceding radical surgical intervention has yielded suboptimal outcomes in terms of DFS when contrasted with the application of cisplatin-based concurrent chemoradiation in the management of LACC [28]. NACT pursued by radical hysterectomy emerges as a compelling alternative to concurrent chemoradiotherapy (CCRT), LDFS is high for individuals with LACC, particularly those presenting with stages IIB and IIIA, which is comparable with stage IB2-IIB pathology [29-31].

In certain special conditions where there is a good objective response after radio or radiochemotherapy and/or NACT, surgical intervention such as hysterectomy with bilateral salpingo-oophorectomy and lymphadenectomy may be opted for. In these patients, if there are risk factors, such as microscopic positive resection margins, and lymphatic, vascular, or perineural invasion on the residual tumor, then this type of treatment becomes a therapeutic option [32]. In the PST group, patients who did not have surgical contraindications underwent surgery. In addition, based on age and personal preference, some patients go through unilateral or bilateral salpingo-oophorectomy and ovarian transposition to the abdominal cavity. At age 35 years or older, smaller tumor size, less advanced stage, lack of nodal metastases, squamous cell histology, objective clinical response, and optimal pathological response are considerable variables for survival of patients who underwent surgery after radial hysterectomy without NACT [29,33,34]. As far as LDFS rates were observed, the rates were 87% in the NACT+ at five years and 100% in the NACT+ surgery group, and the DFS ratio was 89% in the NACT+ surgery group. LDFS rates were reduced in patients in which surgery was done without any NACT (NACT-) group. NACT + surgery is found to be an independent risk prognostic factor for LDFS. Before surgery intervention, DFS survival probability was found to be greater in the NACT - group (p = 0.14) as compared to the NACT- surgery, which is 83% (p = 0.095). The results can be compared through a comprehensive meta-analysis, synthesizing data from six randomized controlled trials (RCTs) encompassing 1,078 subjects grappling with early or locally advanced disease, which unveiled that the adoption of NACT succeeded by radical hysterectomy, which substantially mitigated both the hazard of progression (95% CI = 0.61-0.93, p = 0.008) and the risk of mortality (95% CI = 0.62-0.96, p = 0.02) when juxtaposed with primary radical hysterectomy [25-27,35]. Recent findings indicate that NACT followed by radical surgery results in DFS compared to cisplatin-based CCRT in cases of LACC. The results can be compared to various other studies conducted [35-37]. Our results are also consistent with research in which a meta-analysis was conducted by five RCTs in patients with LACC, which showed better DFS by NACT administration plus radial hysterectomy (95% CI = 0.56-0.82) as compared to definitive radiotherapy without NACT [38]. One limitation of the present study is the limited number of patients, which may not be representative of a larger population of LACC patients in Romania. Another aspect may be that the study may not account for all potential factors such as lifestyle, cardiovascular diseases, and socioeconomic factors. However, it is clear from the present study that NACT can significantly decrease tumor size, stromal invasion depth, parametrial infiltration, lymph-vascular space involvement, and nodal metastases, thus reducing the need for adjuvant radiotherapy.

## Conclusions

NACT in addition to radiation therapy or radiochemotherapy in cases of advanced-stage cervical cancer represents an alternative treatment option compared to the standard treatment, which consists of radiochemotherapy plus brachytherapy. It can be concluded from the present study that in LACC patients, the NACT+-administered group showed good survival probability as compared to the NACT- group. Similarly, when surgery was done in patients who underwent NACT therapy in addition to radiation therapy, it also showed positive results in terms of LDFS and DFS. This type of regimen in certain special conditions shows good objective response after radio or radiochemotherapy and/or NACT; surgical intervention such as hysterectomy with bilateral salpingo-oophorectomy and lymphadenectomy may be opted for. In addition, based on age and personal preference, some patients underwent bilateral or unilateral salpingo-oophorectomy and either bilateral or unilateral ovarian transposition to the abdominal cavity. This may shrink the tumor preoperatively, facilitating its removal and potentially improving survival rates. LDFS was found a better case of surgery done without any NACT treatment. However, this treatment strategy requires further clinical research to produce robust, high-quality evidence. Such studies are necessary to identify specific patient subsets that might benefit most in terms of survival, reduced toxicity, and enhanced quality of life.
